# The pharmacist’s role in the periprocedural management of anticoagulation in Canada: A qualitative study protocol

**DOI:** 10.1371/journal.pone.0353786

**Published:** 2026-07-17

**Authors:** Chioma S. Okpalaora, Stephanie W. Young, Tiffany A. Lee

**Affiliations:** 1 School of Pharmacy, Memorial University, St. John’s, Newfoundland and Labrador, Canada; 2 Pharmacy Program, Newfoundland and Labrador Health Services, St. John’s, Newfoundland and Labrador, Canada; University of South Australia, AUSTRALIA

## Abstract

Anticoagulants are crucial for both the immediate and ongoing management of thromboembolic disorders, including atrial fibrillation and venous thromboembolism, and for patients with mechanical heart valves. However, their management during the periprocedural period requires carefully balancing thromboembolic and bleeding risks, underscoring the need for coordinated and structured periprocedural anticoagulation management (PAM). Pharmacists, with their comprehensive training and expertise in medication management, are well positioned to deliver PAM. However, empirical evidence related to the roles of Canadian pharmacists in PAM, or the barriers, facilitators, and organizational structures that shape their practice is lacking. This protocol paper outlines a qualitative study that uses a qualitative description design grounded in constructionism and naturalism. The study aims to provide an in-depth understanding of pharmacists’ roles and experiences in PAM across Canada, while exploring variations by demographic and practice characteristics. Using purposeful maximum variation sampling, licensed Canadian pharmacists with at least 6 months of current PAM experience will be recruited to take part in one semi-structured interview conducted virtually using Microsoft Teams. Interviews will be recorded and transcribed verbatim and analyzed using conventional qualitative content analysis to inductively generate codes and categories. The findings of this research will address gaps in the literature by providing a detailed description of pharmacists’ roles in PAM and the contextual factors influencing practice across diverse regions and settings in Canada. Findings may inform future PAM service models, professional training, and health system policies to support optimal care coordination and improved patient outcomes in the Canadian anticoagulation care landscape.

## Introduction

Anticoagulants are essential for both the immediate and ongoing management of thromboembolic conditions, including atrial fibrillation, venous thromboembolism, and thrombosis prevention with mechanical heart valves [1]. In North America, anticoagulant interruption in preparation for a procedure or surgery is increasing, with an estimated 250,000 patients undergoing temporary interruption annually [2]. Therefore, the need for optimized and structured periprocedural anticoagulation management (PAM) has become increasingly critical to support patients who require long-term anticoagulation and surgical procedures [3,4].

PAM is a balancing act. The risk for thromboembolism must be balanced against the risk of major bleeding when anticoagulant therapy is dosed close to a surgical procedure [5]. Risk assessment also involves considering patient-specific factors and the type of procedure [5]. In doing so, clinicians often rely on well-recognized, evidence-based guidelines produced by national societies, such as the American College of Chest Physicians, to decide whether to interrupt the anticoagulant, when to stop it, and if bridging therapy is needed [3]. In PAM, bridging therapy refers to the temporary stopping of oral anticoagulants and substituting them with a short-acting agent, usually low molecular weight heparin, to reduce the period in which a patient lacks effective anticoagulation [2].

Pharmacists are well-positioned to lead the delivery of PAM, as their comprehensive training equips them to support the safe and effective use of anticoagulants during the periprocedural period [5]. Pharmacists who work in specialized anticoagulation clinics specifically have been identified by some senior leaders in the field as best equipped to facilitate the development of periprocedural plans for patients due to their expertise in pharmacotherapy, pharmacokinetics, and patient education [4]. Studies from the United States and Australia align with this assertion, indicating that pharmacists working in hospital and ambulatory settings engage in activities such as risk assessment, development of perioperative anticoagulation plans, and bridging protocol management. [4–10] However, based on our review of the literature, there is an absence of empirical evidence describing the role of Canadian pharmacists in PAM and/or the outcomes of patients assigned to pharmacist management. Similarly, barriers and facilitators to pharmacist involvement in PAM have not been studied in the Canadian context. In fact, the only study we could locate related to barriers and facilitators was a recent qualitative study by Capiau et al. (2025) who explored community pharmacists’ involvement with PAM in Belgium; numerous barriers were identified as limiting the pharmacist’s enacted role including, but not limited to, the lack of standardized care protocols, restricted access to medical records, time constraints, and inadequate communication with other healthcare professionals [11].

The lack of Canadian studies is an important gap in the literature that needs to be addressed. Health care delivery systems and pharmacists’ scope of practice in Canada differ from those in the United States, Australia, and Europe, with some Canadian provinces having the broadest scope of pharmacist practice in the world [12]. However, it should be noted that pharmacist scope of practice varies across Canada, as health care delivery remains primarily the responsibility of provincial and territorial governments. Differences in regulatory frameworks, institutional policies, availability of anticoagulation clinics, electronic health record integration, and interprofessional collaboration structures may influence how PAM is organized and who assumes responsibility for decision-making. As a result, pharmacist involvement in PAM may differ substantially across regions and practice settings. To address this knowledge gap, and inform the development of practice and policy, qualitative research in this area is necessary. We know that pharmacists in Canada provide PAM [13,14]; however, their roles, and the barriers and facilitators to these roles, as well as the structure and organization of the PAM services delivered by pharmacists are not well described in the literature. In this paper, we present the protocol for a qualitative study which aims to address this knowledge gap.

The proposed study will provide (i) a detailed description of the pharmacist’s role in PAM as well as barriers and facilitators to the role in Canada and (ii) an in-depth understanding of the structure and organization of PAM services that engage pharmacists in service delivery. The study aligns with existing research on pharmacist-led anticoagulation services and contributes to discussions about pharmacists taking a leading role in the periprocedural management of anticoagulants [15–17]. The findings of this study may inform policy conversations around scope of practice, training programs, institutional policies, interprofessional teamwork, and health care system models to ensure optimal PAM. Findings may also facilitate the development and implementation of standardized care pathways, help to define clear professional responsibilities during management of high-risk medications, and provide strategies to strengthen Canadian pharmacists’ involvement in PAM.

## Materials and methods

### Study aims and objectives

This study aims to explore pharmacists’ involvement in managing anticoagulation for patients undergoing surgical or procedural interventions. The objectives of this study are to:

1. Describe the roles and experiences of pharmacists in managing anticoagulation during the periprocedural period in Canada, including how they engage in providing PAM.
2. Describe barriers and facilitators as well as organizational structures which influence the pharmacist’s ability to provide PAM services in Canada.
3. Describe how pharmacist PAM roles and practices vary across Canada and by other demographic groupings (e.g., gender, practice setting).

### Researcher positionality and reflexivity

The research team comprises a graduate student in pharmacy who trained and practiced as a pharmacist outside of North America as well as two other Canadian clinician researchers with pharmacy practice and health services research backgrounds. The second and last authors (SWY and TAL) have experience conducting qualitative research, including the design and conduct of interview-based studies with pharmacists and the use of qualitative analytic approaches such as content analysis. Their experience will support methodological rigor throughout participant recruitment, data collection, analysis, and interpretation of results.

One team member has experience working as a pharmacist in PAM and anticoagulation management as part of an interdisciplinary team. A second team member works within an anticoagulation management service (AMS) though not directly in the delivery of PAM services. Given the insider perspectives held by team members as pharmacists, two of whom have direct experience in anticoagulation management, we acknowledge that our positionalities and interpretative lenses may influence aspects of data collection and analysis. The highly networked nature of specialized pharmacy practice in Canada may also mean that the two clinician researchers are known to participants. However, it should be noted that neither of these researchers serve in a supervisory or leadership role in this practice area and therefore, the potential for a direct supervisory relationship is not possible. To address and mitigate potential professional biases, we will proactively and systematically engage in reflexivity to enhance confirmability. The following strategies will be implemented: (i) maintaining individual reflexive journals to document personal assumptions, researcher perspectives and evolving insights, and familiarity with participants and its potential influence on data collection and interpretation; (ii) documenting analytic memos during the coding process to capture reflexive thoughts and decision-making rationales; and (iii) conducting regular team meetings to openly discuss potential biases and emerging assumptions to ensure interpretations remain grounded in participants’ data. These reflexive practices will be incorporated into the study audit trail to strengthen the overall trustworthiness of the findings.

### Study design

The qualitative description design described by Sandelowski (2000; 2010) will be used to describe what pharmacists do in providing PAM, how they are involved, and any challenges or gaps in practice from a subjective perspective. Qualitative description aims to produce findings that remain close to the data [18]. This approach is well-suited for research studies like this one, which aim to provide a straightforward, subjective, and low-inference description in everyday language with less theoretical or philosophical involvement; qualitative description is particularly useful in areas where little or no information is known about the topic under investigation [19,20], Furthermore, this qualitative study is grounded in constructionism and naturalism. Constructionism is an epistemological lens which postulates that knowledge, meanings, and realities are co-constructed through interactions, relationships, or context between the participant and researcher. This philosophical perspective acknowledges that knowledge is shaped by the subjective experiences and perspectives of participants rather than an existing objective reality [21,22]. Naturalism is considered the typical theoretical foundation for qualitative description, where sampling, data collection, and data analysis are conducted in their natural state to describe the phenomenon openly and genuinely, without any artificial contributions [22,23].

### Sampling and recruitment

Pharmacists across Canada who are active in managing anticoagulation during the periprocedural period at the time of recruitment will be invited to take part in this study. To be eligible for inclusion, the individual must be a licensed pharmacist in Canada and have at least 6 months of experience in PAM. Pharmacists without direct experience in PAM and/or those who are not currently providing PAM services will be excluded.

Purposive maximum variation sampling will be used. Purposive sampling provides information-rich data that contribute to answering the research questions and developing an in-depth understanding of the phenomenon [19]. Moreover, purposive maximum variation sampling will enable an exploration of the common and unique manifestations in PAM from a diverse perspective of participants capturing role variations based on gender, Canadian region, and practice setting [19]. For example, priority will be given to selecting at least 1 pharmacist from each Canadian province. We will also prioritize capturing experiences based on different practice settings (e.g., interdisciplinary primary care clinic, anticoagulation management service, surgery clinic, community pharmacy, hospital pharmacy). In terms of gender, the selection process will ensure that we hear from women and men, as well as non-binary and transgender pharmacists, where interest is expressed. The experience of providing health care services has been shown in other research studies to differ based on gender and therefore, it is important that we capture the perspectives and experiences of a diverse group of pharmacists [24–26].

Recruitment will involve contacting healthcare institutions, University faculty, professional networks, and associations (e.g., Thrombosis Canada, the Canadian Society of Healthcare-Systems Pharmacy, the Canadian Cardiovascular Pharmacists Network) through their administrative office to disseminate an email invitation and recruitment poster. Recruitment will be managed solely by the first author (CSO) with intentional omission of the clinician researchers’ names from the recruitment materials to minimize potential bias or perceived coercion. The recruitment poster will also be shared across social media platforms (e.g., LinkedIn, Bluesky, Facebook). The email invitation and poster will contain a link or QR code to the screening form within Qualtrics (www.qualtrics.com), the platform used by Memorial University of Newfoundland. The purpose of these screening questions is to confirm eligibility and ensure that maximum variation in participant characteristics is achieved.

Snowball sampling will also be used, as pharmacists providing these specialized services represent a hard-to-reach population; there is no centralized national registry with readily accessible, detailed contact information for research purposes. Given the limited number of licensed pharmacists practicing in this specialized field across Canada, we will leverage the profession’s highly networked nature to expand the sample size, making snowball sampling a suitable method for this study [27]. Study participants will be asked to share names and contact information of potential participants who work in this area with the researcher. Each subsequent participant will, in turn, provide names and contact information of potential interviewees, and so on [28].

The sample size in this study will follow the theory of saturation and the criteria outlined by Sandelowski (1995) for maximum variation sampling. In qualitative research, an adequate sample size balances depth and richness of data, being small enough to allow for detailed, case-oriented analysis, which is central to qualitative inquiry, yet large enough to generate a nuanced and richly detailed understanding of participants’ experiences, reaching theoretical saturation [29]. According to Sandelowski, maximum variation sampling requires the largest minimum sample size among purposeful sampling methods, as greater variability within the study demands more sampling units to adequately explore the phenomenon and reach informational redundancy or theoretical saturation, when additional data no longer yields new insights or themes [29]. We anticipate needing 15–20 participants from across Canada to reach saturation. Saturation will be assessed iteratively through concurrent data collection and analysis. Recruitment will continue until saturation at the point of data collection and analysis is reached, that is until no new codes or categories are identified across interviews, with saturation decisions confirmed through consensus meetings and documented in the study audit trail [29]. Participants will be offered a $75 gift card in appreciation of their time and contribution to this research.

### Data collection

The primary mode of data collection is via in-depth, semi-structured interviews conducted in English using Microsoft Teams. Participant demographic information will be collected as part of the interview to help describe the sample and compare/contrast the experiences of pharmacists by gender, region, and practice setting. Each interview is expected to take 45–60 minutes. The interview will be led by the first author (CSO) using a semi-structured interview guide (see S1 Appendix). One or two other research team members will also be present during the interview to support note taking and triangulation. Following each semi-structured interview, researchers will document contextual details, such as impressions and observable non-verbal cues, as field notes.

With participant consent, the interview will be recorded and transcribed verbatim. CoLoop qualitative data analysis software (www.coloopai.com) will be used to transcribe interview recordings. CoLoop has a transcription verification tool embedded into its program; the first author (CSO) will complete the transcription review using this tool following the Poland transcribing convention [30]. Participants will be provided with a copy of their transcript and given the opportunity to note any errors, omissions, or misunderstandings in the document as well as provide clarifications or remove content if they wish. Any clarifications or corrections received will be incorporated into the final dataset before proceeding with data analysis.

The semi-structured interview guide was developed by the research team and informed by existing PAM literature and the clinical experiences of team members who care for patients taking anticoagulants. Through iterative team discussions, the guide was refined to ensure questions were open-ended and address the study objectives. Probing questions such as “Can you tell us more about that?” will be used to probe deeper into participants’ experiences [31]. To support methodological coherence, a series of three pretest interviews were conducted with local pharmacists who have experience providing PAM to assess and refine the interview guide [32]. After each pretest interview, the research team refined the interview guide based on each pharmacist’s responses and feedback as well as observations about the interview to ensure congruence between the research questions and data collection [32].

### Data analysis

The conventional approach to qualitative content analysis will be used. According to Sandalowski (2000), qualitative content analysis is the preferred data analysis method in qualitative description designs, as it focuses on capturing and summarizing the key informational content of the dataset in a straightforward, data-near manner [19]. Qualitative content analysis is inherently reflexive and interactive, with researchers adjusting their data treatment to integrate new data and insights [19,33]. The conventional method of content analysis follows a naturalistic paradigm, which interprets content or contextual meaning from the data collected; it is particularly suitable when describing a phenomenon for which there is limited research literature or existing theory [33]. This method is also described as inductive, whereby codes, categories, and names for categories are developed directly from participants’ data without imposing preconceived or pre-existing codes, theoretical constructs, or prior assumptions [33].

Analysis will occur concurrently with data collection, allowing emerging insights to inform subsequent interviews [19]. A thorough and repeated reading of all transcripts will be completed to achieve immersion and gain a holistic understanding. Thereafter, two researchers will independently engage in a detailed review of the text to derive codes manually; regular consensus meetings will be held to resolve coding discrepancies. An analysis matrix will be created using Microsoft Word to organize codes and categories across participants. CoLoop qualitative data analysis software will be used as an adjunctive tool to produce summaries and assist with data organization and coding; however, all coding decisions, category development and interpretations will remain grounded in the researcher’s manual coding of the transcripts The coding structure will be iteratively refined as additional transcripts are analyzed and new patterns are identified, which is consistent with the inductive, reflexive nature of conventional qualitative content analysis [33].

Codes will be grouped into broader categories based on their relationships and linkages. Categories and sub-categories will be used to group and organize codes into meaningful clusters in a way that reflects the underlying structure of the data [33]. Further abstraction may be necessary, whereby a large number of subcategories are grouped and organized into a smaller number of main categories with clear definitions developed for each [33,34]. Using the analysis matrix, we will apply the constant comparative approach during coding and category refinement, then conduct cross-case analysis to compare codes and categories within and across key demographic groupings (e.g., gender, practice settings, region); this approach will enable us to identify similarities, differences and patterns in pharmacists’ roles, experiences, and PAM process [19,35,36]. Visual tools such as tree diagrams may also be employed during reporting to illustrate hierarchical relationships among categories [33]. To support findings in the analysis, illustrative excerpts or “exemplars” will be identified from the data for each code or category [33]. In a conventional content analysis approach, theoretical frameworks and previous research are typically integrated later in the process, particularly in the discussion section, to interpret and contextualize the findings [33].

### Data management

All data will be stored electronically within an encrypted, secure OneDrive folder—the cloud-based data storage platform used by Memorial University—and will be accessible only to the research team. Data will be retained for 5 years after publication in accordance with university policy and ethical requirements. All interview recordings will be stored securely and destroyed after they are transcribed and verified for accuracy. The transcript will not contain any identifying information, such as participant name, affiliation, or institution. Any personal identifiers will be removed from the data and replaced with a unique study code and/or pseudonym.

### Rigor and reporting

The study will make use of the four criteria for establishing trustworthiness described by Lincoln and Guba (1985): credibility, dependability, confirmability, and transferability [37,38]. Credibility refers to the truth and interpretation of the data, and whether the data and processes of analysis address the phenomenon under study [38]. This study will make use of member checking to improve the credibility of the study; participants will be given the opportunity to review and confirm the accuracy of their interview transcripts. Credibility will also be enhanced through investigator triangulation, with two researchers independently coding the raw data and examining the same dataset. Findings will be supported by verbatim participant quotations in reporting to further ensure study credibility [23]. Dependability will be achieved through an audit trail; we will document all steps of the research process, including data collection and analysis decisions [23]. A monitoring plan will be created and used during the data analysis process to prevent over-interpretation, ensuring data analysis remains close to participants’ experiences. As Sandelowski (2000) cautions, researchers who have adopted a qualitative description design must avoid going “beyond the data” by making inferences that are not grounded in participants’ actual words or experiences. Detailed descriptions of the research context and participants, as appropriate, will be provided to enable reader assessment of the applicability of the findings to their province/territory or country, thereby supporting the transferability of the study. Employing maximum variation sampling in the recruitment of participants will ensure transferability of the study [23]. The confirmability of the study will be achieved through analytic memos linking interpretations to data, regular consensus meetings, and the use of reflexive journaling to acknowledge researcher biases. All these steps will be taken to ensure the trustworthiness and rigor of the study through naturalistic inquiry [37].

This study will also make use of the consolidated criteria for reporting qualitative research (COREQ) framework [39]. COREQ is a 32- item checklist specifically designed for studies involving semi-structured interviews [39]. COREQ covers 3 main domains of qualitative studies: research team and reflexivity, study design, and analysis and findings. Using this framework will ensure transparent, rigorous, and high-quality reporting of methods and findings, thereby enhancing the trustworthiness and confirmability of the study [39,40].

### Ethical considerations

Ethical approval has been obtained from the Newfoundland and Labrador Health Research Ethics Board (HREB Reference # 2026.007). All participants will receive a copy of the informed consent form via email several days prior to the interview for review. The consent form contains information about the research team, the study’s purpose, procedures, potential risks and benefits, and confidentiality protocols in written form. Informed verbal consent will be obtained from participants before proceeding with the interview or starting the recording. To document the consent process, the lead researcher will include a written statement at the beginning of the document containing each interview transcript confirming that verbal consent was obtained. Confidentiality will be strictly maintained. Interviews will be transcribed verbatim, and the recordings destroyed thereafter; all transcripts will be stored securely in encrypted, password-protected files. All personal identifiers will be removed during transcription, and pseudonyms will be used in reporting findings. Any data that could identify individuals or institutions will be excluded. These steps ensure that ethical principles of autonomy, confidentiality, and the promotion of participant well-being are upheld throughout the research process.

### Study timeline

Participant recruitment will begin during the first week of March 2026 and continue until data saturation is achieved. Data collection is expected to take four or five months, ending by July 31, 2026. Data analysis will commence concurrently with interviews and continue immediately after collection ends. Preliminary findings are anticipated by October 31, 2026, with full results ready for publication by May 2027.

## Discussion

This qualitative study will generate a comprehensive understanding of pharmacists’ roles, experiences, and practice contexts in PAM across Canada. By using maximum variation sampling and in-depth semi-structured interviews, the study will produce detailed descriptions of role enactment, barriers and facilitators, and the structure and organization of PAM services nationally. These findings will address a significant gap in the literature and provide the first Canada‑wide qualitative exploration of pharmacist involvement in PAM.

Existing research highlights the growing recognition of pharmacists’ contributions to anticoagulation care; however, most studies originate from settings outside Canada or focus on general anticoagulation management rather than the periprocedural period. Publications from the United States and Australia demonstrate that pharmacists in PAM participate in risk assessment, protocol development, laboratory monitoring, and interprofessional collaboration [4–10]. A recent qualitative study from Belgium explored community pharmacists’ involvement in perioperative management of antithrombotic agents focusing on facilitators, barriers, and future directions [11]. While focused on community pharmacy settings, the findings highlight that pharmacist expertise remains underused in periprocedural care and that structural and communication barriers limit their potential contributions.

The qualitative study proposed here has several strengths that highlight its importance. A major strength lies in the adoption of the qualitative description design described by Sandelowski, which will provide a rich, straightforward description of pharmacists’ roles in PAM in Canada using terminology that stays close to the data. This approach minimizes researcher interpretation and interference, providing a subjective view through the pharmacist’s lens. Data collection occurs in a naturalistic manner through in-depth, semi-structured interviews with pharmacists who have direct experience managing anticoagulation during the periprocedural period. By using this design, this study will capture detailed findings of pharmacists’ roles, experiences, facilitators, barriers, and everyday practices without imposing preconceived ideas, thereby enhancing the credibility and relevance of the results to inform clinical practice and policy development. This will be the first qualitative study in Canada to provide an in-depth understanding of this topic, making it one of the key strengths of this study. By using maximum variation sampling, this study is designed to capture and highlight variations in pharmacist practice across Canada in delivering PAM. This study also addresses a gap in the literature by providing a comprehensive and detailed description of the organization and structure of PAM services that engage pharmacists as core team members.

Another notable strength of this study is the use of several qualitative rigor strategies. Independent coding by two researchers, followed by consensus discussions, reduces professional bias and ensures the credibility of this study. Concurrent data collection and analysis will facilitate the refinement of interview questions and the identification of data saturation, supporting the depth and richness of the findings. The use of verbatim participant quotes, documented field notes, and member checking in this study promotes transparency, accuracy, and consistency between participants’ data and the reported findings. All these strategies will position our study as a robust, credible contribution to the Canadian pharmacy literature on PAM.

This study also has some limitations. First, the study will focus on Canadian pharmacists with experience in managing anticoagulation during the periprocedural period. Findings may not be transferable to pharmacists practicing outside Canada or to other healthcare professionals involved in PAM. Second, variations in practice settings, institutional protocols, regional healthcare structures, and scope of practice across Canada may limit the transferability of findings, as the experiences described may be context specific. Lastly, recognizing that there are two official languages in Canada, the focus of the interview on English-speaking pharmacists potentially excludes francophone pharmacists who prefer to communicate in French. This may reduce representation from French-speaking regions or practice settings and may affect the transferability of the findings to these regions in Canada.

By exploring facilitators, barriers, and opportunities for improvement in the delivery of PAM by pharmacists, this study will generate recommendations and practical insights that may expand pharmacists’ roles, optimize clinical practice during periprocedural care, and inform pharmacist training and education. To inform practice and policy, study findings will be disseminated through publication in peer-reviewed journals relevant to pharmacy practice, as well as presented at national or international conferences. Findings may also be presented as webinars and/or continuing education activities for pharmacists. Other presentations to healthcare staff and administrators, as well as publications in relevant organizational and professional society newsletters, may take place.

## Supporting information

S1 Appendix Interview guide. (DOCX)
