# Supplementary material for: The pharmacist’s role in the periprocedural management of anticoagulation in Canada: A qualitative study protocol
Source: PLoS One. 2026 Jul 17;21(7):e0353786. doi: 10.1371/journal.pone.0353786 (PMC13379006; doi:10.1371/journal.pone.0353786)
Supplement: S1 Appendix — (DOCX) [file pone.0353786.s001.docx]

**S1 Appendix: Interview Guide**

**Part 1: Preamble**

We are interested in learning more about the role of pharmacists in periprocedural anticoagulation management, which we refer to as PAM. The aim of this qualitative study is to provide a detailed description of the pharmacist’s role in PAM across Canada and to gain an understanding of the structure and organization of PAM processes which influence their ability to provide optimal anticoagulation therapy in patients requiring surgery/procedure.

This interview will be recorded to make sure we are accurate in capturing your experiences and to enable transcription. The recording will be stored securely and destroyed after the interview has been transcribed and verified for accuracy. Identifying information, such as your name, affiliation, institution, or so on, will be removed from the transcript and replaced with a unique study code or pseudonym. So, feel free to express yourself; be candid, frank, and honest.

The transcript will be sent to you for review; at that time, we ask that you identify any errors, omissions, or misunderstandings in the document as well as any content that you wish to have removed. Transcript review is not mandatory. However, it does help to improve the credibility of the research and confirm the accuracy of your interview transcripts.

The other researcher(s) present today [researcher names] are here to support note taking and to observe this interview. They might chime in from time to time if they have questions for you.

Do you have any questions about the consent form or study before we begin?

Do you consent to taking part in this interview and to interview being recorded?

[If yes to verbal consent] Ok. Thank you. I will document your verbal consent on the transcript of your interview.

[Start recording] Ok. Let’s get started.

**Part 2: Interview Questions**

1. Can you tell us about your specific role in managing anticoagulation for patients undergoing surgery or procedures?

*Possible Prompts:*

- *Are you able to make decisions about patient care independently or is your role dependent on working with another provider who has the authority to sign off on decision-making?*
- *Do you utilize pharmacist* *prescribing to provide PAM?*
- *Are there specific agreements, policies, or protocols in place which guide your practice? If so, can you share the document or protocol that is used?*
- *Do you have any training or certification in anticoagulation management?*
- *Do you have any training specifically in periprocedural anticoagulation management?*
- *Are there other provider types that you collaborate with to provide PAM?*
- *How many hours* ***per week*** *do you work providing PAM?*

1. Thinking about your practice specifically, can you describe a typical patient case that you have been involved with?

*Possible Prompts:*

- *In the example you gave, the patient was taking [state drug class]. Do you only manage patients taking that drug class? If not, can you tell us about other anticoagulants that you manage?*
- *In the example you gave, the patient had [state indication]. Is this the only type of patient case that you would manage? If not, what other types of indications do you manage within your practice?*
- *Can you describe the processes you follow to deliver PAM? For example, can you* *walk us through exactly what you would do in managing that patient?*
- *After you provide the patient with a plan or implement a PAM protocol, do you have any further contact with the patient? Or is that the point in time at which your care for that patient ends*
- *What mode of delivery (e.g., in-person, telephone, virtual care) do you most commonly used to provide PAM? Are the other modes of delivery ever used? Or have they been used in the past?*

1. Thinking about your own practice, how do you typically become involved in managing periprocedural anticoagulation for a patient? For instance, do you receive referrals for certain patients or procedures, or do you identify these patients yourself?
2. How is the [clinic, pharmacy, or service] where you provide PAM structured, and how does it operate?

*Possible Prompts:*

- *Who are the other members of your team?*
- *Regarding the other team members, what is their role or designation?*

1. What has helped you in providing periprocedural anticoagulation management? By this, I mean what has supported or helped to facilitate your involvement in PAM.

*Possible Prompts:*

- *Professional practice support?*
- *Financial support?*
- *Educational* *support?*
- *Departmental support?*

1. What barriers have you encountered in providing periprocedural anticoagulation management?

*Possible Prompts:*

- *[If discusses barriers within the clinic or practice environment] Anything beyond the clinic environment that may have been a barrier?*
- *Scope of practice barriers?*
- *Institutional barriers?*

1. How would you describe your overall experience in managing anticoagulation during the periprocedural period?
2. What changes, if any, would you like to see to improve or expand your involvement in PAM?
3. What else would you like to tell us about your role in PAM or providing care to patients in this manner that we have not given you an opportunity to say?

**Part 3: Demographic Questions**

**Before we wrap up today, I have a couple of questions about you and your practice.**

1. What is your gender?
2. What province do you work in?
3. How many years have you been practicing as a pharmacist?
4. How many years have you been practicing as a pharmacist who provides PAM?
5. What is your current title or position?

**Part 4: Snowball Sampling Question**

We’re hoping to speak with other pharmacists with experience in managing anticoagulation during the periprocedural period. In keeping with our participant referral process, which is referred to as snowball sampling, we are wondering if you might know any pharmacists or organization (with pharmacists as the core team member) across Canada working in this area who we should invite to take part in this study.

[If yes], Are you comfortable sharing their name and contact information (email or practice site location) with us? Please note that we will not disclose who provided the referral, as your privacy as a participant in this study is of utmost importance. Maintaining the confidentiality of our research participants is also an important measure to ensure the integrity of the research process.
